# Primary isolated asymptomatic gastric tuberculosis of the cardia mimicking gastric stromal tumor: a rare case report and literature review

**DOI:** 10.1186/s12876-020-01242-x

**Published:** 2020-04-15

**Authors:** Mingnan Lv, Kejiang Tang, Yajie Meng, Chuan Tian, Min Wang

**Affiliations:** Department of Gastroenterology, The People’s Hospital of Nanchuan, No. 16 South Street, Nanchuan District, Chongqing, 408400 China

**Keywords:** Gastric tuberculosis, Gastric stromal tumor, Endoscopic submucosal dissection, Case report

## Abstract

**Background:**

Primary isolated gastric TB of the cardia presenting as a submucosal tumor is extremely rare.

**Case presentation:**

A 60-year-old female was admitted to our department; endoscopy revealed a smooth protruding lesion in the gastric cardia. The patient was diagnosed with a gastric cardia stromal tumor and the lesion was seen in muscularis propria by endoscopic ultrasonography (EUS). Endoscopic submucosal dissection (ESD) revealed that the lesion was filled with a milky, white liquid and white granulation tissue. Acid-fast specimen staining was negative. Hematoxylin and eosin staining showed patches of caseating necrosis and granulomatous inflammation. Gene sequencing subsequent to polymerase chain reaction (PCR) analysis of the ESD specimen identified *Mycobacterium tuberculosis* (M. TB) DNA fragments. The patient was put on ATT for 6 months.

**Conclusion:**

Primary isolated gastric TB of the cardia should be suspected in patients without clinical symptoms whose manifestations are similar to those associated with submucosal tumors. TB-PCR may be helpful for further diagnosis.

## Background

Gastric TB is very rare, regardless of primary or secondary infection. Clinically, gastric tuberculosis often resembles peptic ulcer disease, cancer or lymphoma under endoscopy [1, 2]. There have been several reported cases of gastric TB mimicking a subepithelial tumor or gastrointestinal stromal tumor, but most cases were mainly located in the antrum of the stomach [3–6]. Gastric tuberculosis in the cardia of the stomach usually appears like a gastric stromal tumor [4]. To our knowledge, it is extremely rare. Approximately 20 years, only three cases have been reported. Here, we report a case of gastric cardia TB that manifested as a gastric stromal tumor without evidence of pulmonary involvement or other syndromes, except for a BMI < 18 kg/m^2^.

## Case presentation

**Fig. 1:**
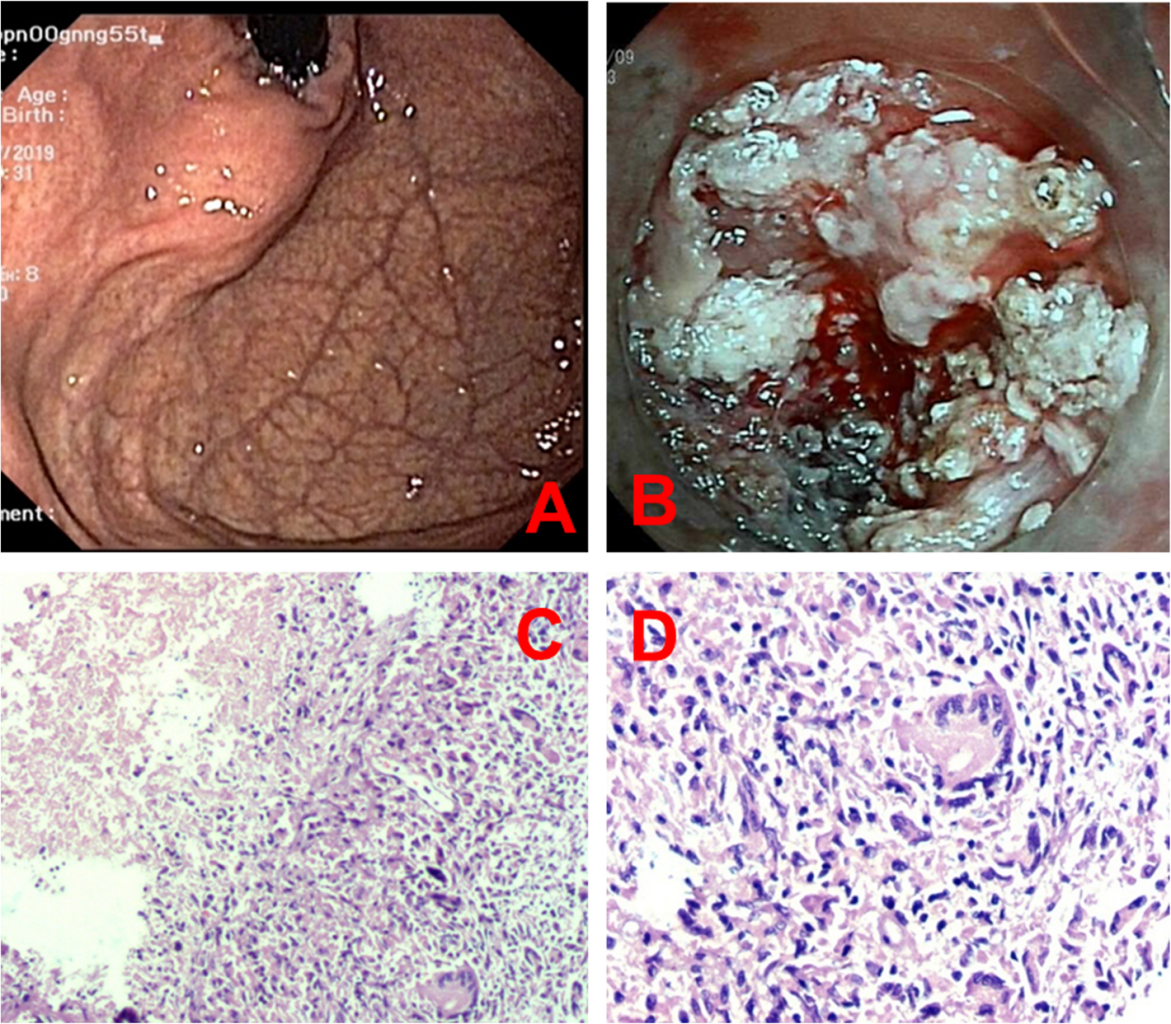
Images of endoscopy and staining. **a**, Endoscopy revealed a smooth protruding lesion in gastric cardia; **b**, During the ESD, the lesion was observed to originate from the fourth layer and was filled with a milky, white liquid and white granulation tissue; **c** and **d**, Images of Hematoxylin & Eosin staining showing patches of caseating necrosis, Langhans giant cells and granulomatous inflammation (C: 100x, D: 200x)

A 60-year-old female was admitted to our gastroenterology department; endoscopy revealed a smooth protruding lesion in the gastric cardia in another hospital (Fig. 1a). The mass was not soft when pressed with forceps and had a negative rolling sign. After admission, the results of other physical examinations were unremarkable, except for the body mass index, which was 16.44 kg/m^2^ (weight 37 kg, height 1.50 m). The patient did not present with abdominal pain, fever, cough, expectoration, hemoptysis, swallowing difficulties or diarrhea. The patient did not have any history of chronic or significant medical or family illnesses. After admission, routine laboratory tests did not indicate any abnormalities.

**Fig. 2:**
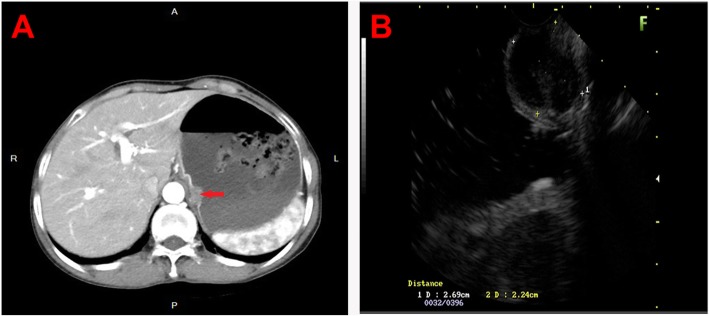
Images of the case with CT and EUS. **a**, Contrast-enhanced abdominal CT revealed a 2.0-cm relatively well-defined soft tissue mass (red arrow) on the small curved side of the cardia; **b**, Linear EUS examination revealed a hypoechoic lesion that originated from the fourth layer, with an uneven internal echo and a clear boundary

Contrast-enhanced abdominal CT revealed a 2.0-cm relatively well-defined soft tissue mass on the small curved side of the cardia (Fig. 2a). There was no enlarged lymph nodes in the upper abdomen. In addition, the CT scan of the chest was normal.

At admission, the linear EUS examination revealed a hypoechoic lesion that originated from muscularis propra, with dimensions of 22 mm × 17 mm and an heterogeneous echo and clear boundary (Fig. 2b). According to EUS, it was diagnosed as a gastric cardia stromal tumor.

The patient was originally suspected of having a gastric stromal tumor. We performed ESD. During the operation, it was observed that the lesion was filled with a milky, white liquid and white granulation tissue (Fig. 1b), suggesting that it was likely not a gastric stromal tumor. Therefore, we resected all the granulated tissue. The pathology results of the ESD specimen were negative for acid-fast staining. Hematoxylin and eosin staining showed patches of caseating necrosis and granulomatous inflammation (Fig. 1c and d). Gene sequencing subsequent to PCR analysis of the ESD specimen identified *Mycobacterium tuberculosis* (M. TB) DNA fragments.

The patient received ATT consisting of 2HREZ/6HR regimen of isoniazid 5 mg/kg once daily, rifampicin 10 mg/kg once daily, ethambutol 15 mg/kg once daily, and pyrazinamide 25 mg/kg daily for the initial 2 months, followed by isoniazide and rifampicin at the same dosages for the next 4 months. The patient’s liver and kidney function was closely monitored for the first week, and it appeared normal. The patient was followed monthly, and no side effects of the ATT drugs were observed. After 6 months of treatment, she had gained 5 kg, and her appetite had improved; she was feeling healthy. We will continue to closely monitor the patient’s liver and kidney functions.

## Discussion and conclusions

Abdominal TB is the third most common extrapulmonary manifestation of TB. The gastrointestinal tract is the sixth most common site of extrapulmonary TB, and the ileocecal region is the most common site [7, 8]. The unique characteristics of the stomach, such as the presence of gastric acid, rapid gastric emptying, and the scarcity of lymphatic tissue in the gastric wall, may protect the organ from TB [9].

According to previously published case reports and series, gastric TB can be classified into four types: primary gastric TB, gastric TB secondary to pulmonary TB, gastric TB associated with the involvement of other parts of the gastrointestinal tract, and gastric TB in HIV-positive patients [10]. The routes of TB infection in the stomach are unclear [11, 12], and the main causes of isolated or primary gastric TB may be the ingestion of unpasteurized milk infected with bovine TB or a severely immunocompromised condition [13, 14].

The most common site of gastric TB is the antrum region, especially the lesser curvature near the prepyloric region [3–6, 15]. Additionally, this is the most common site for peptic ulcers, thus resulting in a mucosal breach at this site. TB reaches the stomach following a mucosal breach due to ulcers, gastritis damage, erosions or ecchymosis. Primary isolated gastric TB is a rare occurrence, especially without evidence of TB elsewhere. To our knowledge, for nearly 20 years, there were only two cases of isolated cardia TB, and only one case of fundus TB was reported [16–18]. Our case involved isolated TB in the gastric cardia without any other syndromes, except for a BMI < 16.44 kg/m^2^**.** The patient did not have pulmonary TB or any other immunodeficiency diseases. During the ESD procedure, we found that the lesion originated from the intrinsic muscle layer but did not breach the serosa layer. Therefore, we misdiagnosed the lesion as a gastric stromal tumor. In 2012, Vishal Gupta reported primary gastric TB mimicking a gastrointestinal stromal tumor in the gastric antrum. On EUS, the lesion appeared as a round, well-defined, uniformly hypoechoic lesion occupying the fourth layer of the gastric wall, with no significant perigastric lymphadenopathy [5]. In our case, the intraoperative and EUS manifestations were similar. Therefore, we believe that the manifestations of isolated gastric TB on EUS are similar to those of gastric stromal tumors or leiomyomas, which are hypoechoic or nearly nonechoic. Most of the lesions originate from the fourth layer of the gastric wall. Therefore, it was easy to misdiagnose as a gastric submucosal tumor. However, during the ESD, some milky, white secretion outflow was observed, and the peripheral tissue presented with white granulation. It was necessary to be aware of TB, especially if the patient has a history of TB or immune deficiency. In the clinic, there are various manifestations of gastric TB, such as SMT, gastric carcinoma, gastric ulcers, diffuse gastric mucosal lesions, etc. The gold standard to determine the pathology is endoscopic guided biopsy, especially combined with acid-fast staining; TB-PCR, PET-CT and EUS-guided biopsy are also helpful in the diagnosis of gastric TB and its differentiation from gastric stromal tumor and gastric cancer [2, 11, 19, 20].

We believe that the main manifestation of isolated gastric TB of the cardia on EUS is origination from the fourth layer of the gastric wall, similar to a gastric stromal tumor. During the ESD, there was some milky, white secretion outflow, and the peripheral tissue presented with white granulation. It was necessary to be aware of TB. TB-CRP may be helpful for further diagnosis. Gastric TB is rare, with only sporadic reports and various manifestations. The mechanism of isolated TB of the cardia or fundus of the stomach is unknown. In patients without clinical symptoms, the manifestations may be similar to those of submucosal tumors. Even when combined with endoscopy, EUS, CT and other examinations, achieving an accurate preoperative diagnosis is still challenging and requires further study.

## Data Availability

All data analyzed during this study are included in this published article.

## Abbreviations

TB: Tuberculosis
M. TB: *Mycobacterium tuberculosis*
EUS: Endoscopic ultrasonography
ESD: Endoscopic submucosal dissection
PCR: Polymerase chain reaction
